# Analysis of Zinc-Exporters Expression in Prostate Cancer

**DOI:** 10.1038/srep36772

**Published:** 2016-11-11

**Authors:** Chandra K. Singh, Kareem M. Malas, Caitlin Tydrick, Imtiaz A. Siddiqui, Kenneth A. Iczkowski, Nihal Ahmad

**Affiliations:** 1 Department of Dermatology, University of Wisconsin, Madison, WI 53706, USA; 2 Department of Pathology, Medical College of Wisconsin, Milwaukee, WI 53226, USA; 3 William S. Middleton VA Medical Center, Madison, WI 53706, USA

## Abstract

Maintaining optimal intracellular zinc (Zn) concentration is crucial for critical cellular functions. Depleted Zn has been associated with prostate cancer (PCa) progression. Solute carrier family 30 (SLC30A) proteins maintain cytoplasmic Zn balance by exporting Zn out to the extracellular space or by sequestering cytoplasmic Zn into intracellular compartments. In this study, we determined the involvement of Zn-exporters, SLC30A 1–10 in PCa, in the context of racial health disparity in human PCa samples obtained from European-American (EA) and African-American (AA) populations. We also analyzed the levels of Zn-exporters in a panel of PCa cells derived from EA and AA populations. We further explored the expression profile of Zn-exporters in PCa using Oncomine database. Zn-exporters were found to be differentially expressed at the mRNA level, with a significant upregulation of SLC30A1, SLC30A9 and SLC30A10, and downregulation of SLC30A5 and SLC30A6 in PCa, compared to benign prostate. Moreover, Ingenuity Pathway analysis revealed several interactions of Zn-exporters with certain tumor suppressor and promoter proteins known to be modulated in PCa. Our study provides an insight regarding Zn-exporters in PCa, which may open new avenues for future studies aimed at enhancing the levels of Zn by modulating Zn-transporters via pharmacological means.

Worldwide, PCa is the second most frequently diagnosed cancer with 1.1 million new cases estimated to have occurred in 2012, and fifth-leading cause of cancer death in males^1^. According to estimates from the American Cancer Society, in the United States 180,890 new cases of PCa are expected to be diagnosed and 26,120 patients are expected to die from this disease in the year 2016^2^. The existing treatments, as well as surgical approaches, have not been fully effective either for prevention or treatment of PCa. While PCa affects men of all races, the incidence and mortality rates in men of African origin, regardless of where they live, are significantly higher than those of other ethnicities. SEER (Surveillance, Epidemiology, and End Results) data shows a higher PCa incidence in AA men (~2.5 times) compared to EA, both in terms of age of onset, morbidity, and presentation with advanced cancer^3^. Even after adjusting for demographic, socioeconomic, clinical, and pathologic factors, the risk for PCa remained statistically higher for AA men^4^. Thus, there is an urgent need to understand the critical determinants of PCa development and progression, as well as the causes of the racial disparity, and to ultimately identify specific molecular target(s) in order to devise mechanism-based approaches for the management of PCa.

Zn, the second-most abundant trace element in the human body, has been shown to be essential for ~300 different cellular processes^5^. Studies have shown that Zn plays a critical role in a number of prostatic functions, including citrate production and sperm health. Specifically, human prostate cells accumulate several times more Zn than other soft tissues (~150 μg Zn/g compared with ~20–50 μg Zn/g)^5^. Zn is required to maintain a metabolic situation that is unique to the prostate and is characterized by the production and secretion of high amounts of citrate. In the healthy prostate, high Zn concentrations inhibit a mitochondrial aconitase enzyme, leading to the truncation of the Krebs cycle at the first step of citrate oxidation, thereby triggering high citrate levels in the prostatic fluid, which is an important constituent of semen. This is an energy-inefficient process, and prostate cells spend an enormous amount of energy to achieve this task. During neoplastic transformation, the normal prostate epithelial cells are metabolically transformed into citrate-oxidizing cells that lose the ability to accumulate Zn^6^, which allows them to accumulate energy that can be used for cancer growth and metastasis. Studies have shown that in cancerous prostatic tissue, the Zn level is significantly diminished (~85% *versus* normal tissue)^7^,^8^. Further, intracellular Zn levels have a strong inverse correlation with PCa progression^6^. Even significant lower concentrations of serum Zn has been noticed in PCa patients compared to normal controls (reviewed in ref. 9). It is not clear whether low Zn content is a cause or result of carcinogenesis. However, recent data suggest that it may be the former, and Zn is definitely a critical factor in PCa progression (reviewed in ref. 5).

Although a number of proteins are known to regulate cellular Zn homeostasis, the most prominent are two protein families of Zn transporters: solute carrier family 39 (SLC39A) and solute carrier family 30 (SLC30A). These protein families with opposing functions regulate Zn influx and efflux in the cell as well as intracellular compartments such as vesicle, endoplasmic reticulum, Golgi apparatus and mitochondria. The ten members of the SLC30A family, also known as Zn-exporter proteins, reduce the cytoplasmic Zn concentration by transporting it out of the cell or into organelles, thus preventing Zn toxicity. They are located in the cell membrane as well as in membranes of the endoplasmic reticulum, mitochondria, Golgi and vesicle^10^. Though Zn depletion is a well-established phenomenon in PCa, only scarce information on Zn-exporters (SLC30A 1–10) is available (reviewed in refs 10 and 11). In this study, we determined whether differential modulation of Zn-exporters is a critical determinant in PCa.

## Results and Discussion

This study was designed to determine the expression profile of Zn-exporters, SLC30A 1–10 in PCa cell lines and tissue samples obtained from AA and EA populations. We used 12 PCa tissue and 12 benign (matched adjacent prostate) tissue samples from AA patients (in duplicate) and 13 PCa tissue and 13 benign (matched adjacent prostate) tissue samples from EA patients (in duplicate). For combined analysis where the samples were examined for total PCa vs benign (EA + AA), as well as for tumor stage, and grade-wise analysis, we also included 1 benign and 1 tumor sample each from an Asian and a Hispanic population. The inclusion of Asian and Hispanic samples with EA and AA samples did not change the overall analysis but increased the total number of samples (n). There were 18 PCa samples for stage II, and 9 for stage III. Using prognostic grade groupings^12^, group 1 (i.e. 3 + 3) had n = 7, group 2 (i.e. 3 + 4) had n = 11, group 3 (i.e. 4 + 3) had n = 7, and group 5 (i.e. 4 + 5 or 5 + 4) had n = 2.

### qRT-PCR analysis of Zn-exporters

In the analysis of Zn-exporters SLC30A 1–10, each adjacent benign sample served as an independent reference control for their respective tumor samples; hence, benign samples between EA and AA are not comparable. Therefore, it cannot be concluded that there is no difference in Zn-exporters of benign tissues of AA and EA. Further, in the analysis of *in vitro* data, all PCa cell lines were compared to the NrPEC cell line. The RWPE1 cell line has been used as an additional normal prostate reference sample. For some genes, RWPE1 cells did not show the same pattern of expression of Zn-exporters as for NrPEC. This may be because RWPE1 cells, though derived from the peripheral zone of histologically normal adult human prostate cells, are a virally-immortalized epithelial line.

#### SLC30A1

SLC30A1, also known as ZnT1, is widely expressed in human tissues and primarily localized to the plasma membrane. It contributes to cytoplasmic Zn balance by exporting Zn to the extracellular space^13^. Specifically, SLC30A1 is typically found on the basolateral membrane of polarized epithelial cells, a property that facilitates Zn absorption in the intestines and Zn reabsorption in the kidneys^14^,^15^. However, SLC30A1 is located on the apical membrane in pancreatic acinar cells which may enable pancreatic Zn secretion^16^. SLC30A1 mRNA expression is partly regulated by cellular Zn levels^17^, which help to protect the cell from intracellular Zn toxicity.

**Figure 1:**
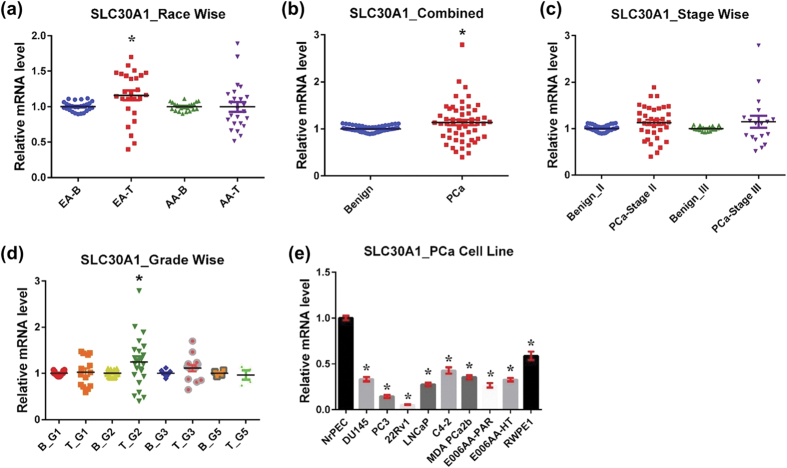
SLC30A1 in PCa. SLC30A1 mRNA levels were analyzed using qRT-PCR assays, followed by statistical analysis using GraphPad Prism 5 software as detailed in ‘Materials and Method’ section. **(a)** Race-wise analysis of SLC30A1 in PCa. Samples are represented as EA-B, benign prostate from European American (n = 13); EA-T, prostate tumor from European American (n = 13); AA-B, benign prostate from African American (n = 12); AA-T, prostate tumor from African American (n = 12). **(b)** SLC30A1 in combined PCa samples, which includes all samples (27 benign and 27 tumor samples). **(c)** Tumor stage-wise analysis of SLC30A1 in PCa (n = 18 for stage II and 9 for stage III, adjacent benign and tumor samples). **(d)** Tumor grade-wise analysis of SLC30A1 in PCa (n = 7 for grade group 1, 11 for group 2, 7 for group 3, and 2 for group 5, adjacent benign and tumor samples). **(e)** SLC30A1 in PCa cell lines representing data of 3 biological replicates. The statistical analysis was done using one-way analysis of variance (ANOVA) followed by Sidak’s multiple comparison tests for data presented in (**a,c,d**), and Dunnett’s multiple comparison tests for data presented in (**e**). Two-tailed unpaired t-test was done for data presented in (**b**). All data are expressed as mean ± standard error and statistical significance are denoted with *p-value < 0.05.

In this study, we observed a significant upregulation of SLC30A1 mRNA in PCa tissue, compared to benign tissue in EA population (Fig. 1a). However, the AA population showed no difference in SLC30A1 expression between benign and cancer tissues, suggesting a racially differential status for SLC30A1. Moreover, in the combined analysis (EA + AA), SLC30A1 overexpression remained statistically significant (Fig. 1b). The SLC30A1 upregulation was not found to correlate with tumor stage as it remained the same in stage II and III samples (Fig. 1c). By tumor grade-wise analysis, the significant increase in SLC30A1 was noticed only in grade group 2 cancer samples (Fig. 1d). Surprisingly, SLC30A1 mRNA expression was significantly downregulated in all tested PCa cells irrespective of whether derived from EA or AA (Fig. 1e).

The absence of SLC30A1 in mice is known to be embryonically lethal^18^. In a study by Hasumi et al, SLC30A1 mRNA level was found to be significantly lower in PCa tissues compared to benign hyperplastic tissues^19^. Interestingly, SLC30A1 is the only member of the SLC30 family that exports cytoplasmic Zn ions across the cell membrane to the extracellular space. The human tissue data of this study suggest that Zn might be exported uncontrollably, and that might lead to Zn depletion in the prostate. However, it is yet to be determined whether prostate cells lose the ability to accumulate Zn, or export Zn excessively out of the cells, or both.

#### SLC30A2

SLC30A2, also known as ZnT2, is localized to vesicular compartments, mainly endosomes and lysosomes^20^. SLC30A2 reduces cytosolic Zn levels by transporting Zn from the cytosol into the lumen of vesicles that contain SLC30A2. SLC30A2 activity is critical for enriching breast milk with Zn during lactation^21^. Furthermore, SLC30A2 null mice exhibited irregular mammary gland architecture and function^22^. These findings implicate SLC30A2’s prominent role in breast health. In rat prostate, both SLC30A2 expression and Zn concentration in the dorsolateral lobes were higher than in the ventral lobes^23^. The expression of SLC30A2 mRNA in the ventral prostate of aged rats was 21-fold higher than in the ventral prostate of young rats, suggesting SLC30A2 expression in the prostate may be correlated with age^24^.

In this study, although the SLC30A2 expression shows some differences in PCa between EA and AA population, as well as in tumor stage and grade, this remains inconclusive (Supplemental Fig. 1a–d). This is because SLC30A2 was not detectable in most of the human PCa tissue samples. The data presented are those samples where it was detectable. This may have happened due to negligible expression of SLC30A2, as is known for tissue-restricted expression in mammals. Interestingly, in the *in vitro* study, we found a significant decrease in SLC30A2 mRNA expression in all the PCa cell lines tested here except C4-2. (Supplemental Fig. 1e). Contrarily, C4-2 demonstrated overexpression of SLC30A2.

#### SLC30A3

SLC30A3, also known as ZnT3, serves to transport Zn into the pre-synaptic vesicles to be later released during neuronal transmission^25^,^26^. SLC30A3 is also expressed in pancreatic β-cells and plays a role in insulin production^27^. SLC30A3 expression decreases with older age^28^ and is downregulated in patients with Alzheimer’s and Parkinson’s diseases^29^. This protein has also been implicated in diabetes, and knockdown of SLC30A3 in INS-1E cells (a pancreatic β-cell model) resulted in significant reduction in insulin secretion^30^. Iguchi and colleagues have compared the expression of SLC30A1 and SLC30A3 between androgen-responsive LNCaP cells and its androgen-independent subline, AIDL cells, and found that AIDL cells exhibited a lower level of SLC30A1 and a higher level of SLC30A3, suggesting that SLC30A3 expression undergoes an androgenic regulation in LNCaP cells^31^.

In this study, we did not find a detectable level of SLC30A3 in most of the human PCa samples. However, in some of the samples where it was detectable, we found a significant decrease in SLC30A3 mRNA expression in PCa tissue for both EA and AA populations (Supplemental Fig. 2a,b). Similarly, by stage- and grade-wise analysis, though inconclusive, SLC30A3 seems to decrease in PCa progression (Supplemental Fig. 2c,d). On the contrary, SLC30A3 mRNA expression was significantly high in most of the PCa cell lines (Supplemental Fig. 2e).

#### SLC30A4

SLC30A4, also known as ZnT4, is commonly localized to the *trans*-Golgi network and cytoplasmic vesicles^32^. In response to Zn treatment, SLC30A4 relocalizes from the Golgi apparatus to an intracellular compartment^33^. SLC30A4 influences cytosolic Zn homeostasis by translocating cytoplasmic Zn to lysosomes^34^ and depositing cytoplasmic Zn into secretory vesicles in lactating mammary glands^35^. SLC30A4 is abundantly expressed in the brain, small intestine, and mammary gland^32^. Mutations in SLC30A4 are responsible for *lethal milk* (lm) mouse, a condition resulting in low Zn in milk^32^. However, this relationship between SLC30A4 and Zn levels in mouse milk does not correlate with Zn deficiency in human milk^36^. Henshall *et al*. have shown that SLC30A4 was significantly overexpressed in PCa compared to normal tissues from other organs, but decreased during progression from localized cancer to invasive cancer^37^.

**Figure 2:**
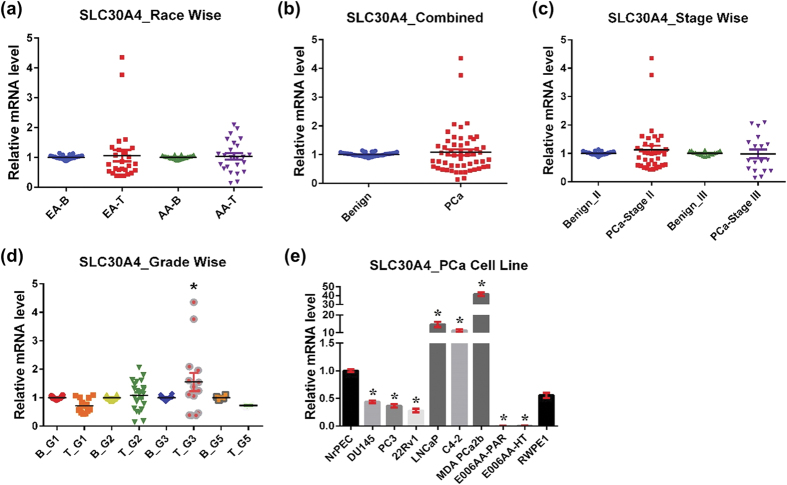
SLC30A4 in PCa. SLC30A4 mRNA levels were analyzed as detailed in Fig. 1. (**a**) Race-wise analysis of SLC30A4 in PCa. **(b)** SLC30A4 in combined PCa samples. **(c)** Tumor stage-wise modulation of SLC30A4 in PCa. **(d)** Tumor grade-wise modulation of SLC30A4 in PCa. **(e)** SLC30A4 in PCa cell lines.

Interestingly, we found no significant difference in SLC30A4 mRNA expression between PCa tissue and benign tissue for either racial population (Fig. 2a). There was no change in SLC30A4 expression in combined PCa analysis and in the analysis of tumor stage and grade, except for cancer grade group 3, which was upregulated (Fig. 2b–d). SLC30A4 was downregulated in most PCa cell lines except LNCaP, C4-2, and MDA PCa 2b, which are known to be derived from a metastatic site (Fig. 2e). In these three PCa cell lines, SLC30A4 was significantly overexpressed.

#### SLC30A5

SLC30A5, also known as ZnT5, is primarily associated with the Golgi apparatus and secretory granules, and is expressed in many different tissues, especially the pancreas, kidney, and liver^38^. SLC30A5-mediated transportation of cytosolic Zn into the secretory pathway is critical for the homeostatic maintenance of secretory function in cells^39^. Furthermore, SLC30A5 is important for depositing Zn into secretory granules in pancreatic β cells^38^. Uniquely, SLC30A5 heterodimerizes with Zn transporter SLC30A6 in the early secretory pathway and loads Zn onto Zn-requiring enzymes involved in secretory pathway function^40^. SLC30A5 deficient mice show poor growth and reduced bone density due to impaired osteoblast maturation to osteocytes^41^. Disruption of SLC30A5 can cause male-specific cardiac death in mice due to bradyarrhythmias^41^. Additionally, SLC30A5 is required for contact hypersensitivity and mast cell-mediated delayed-type allergic responses^42^.

**Figure 3:**
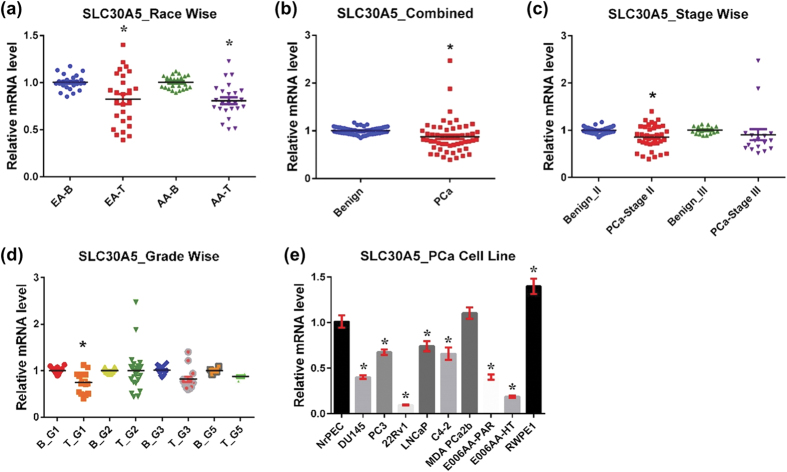
SLC30A5 in PCa. SLC30A5 mRNA levels were analyzed as detailed in Fig. 1. (**a**) Race-wise analysis of SLC30A5 in PCa. **(b)** SLC30A5 in combined PCa samples. **(c)** Tumor stage-wise modulation of SLC30A5 in PCa. **(d)** Tumor grade-wise modulation of SLC30A5 in PCa. **(e)** SLC30A5 in PCa cell lines.

Our study shows that SLC30A5 mRNA expression was downregulated in PCa tissue in both EA and AA populations, as well as in combined PCa analysis (Fig. 3a,b). Our analysis further shows that SLC30A5 downregulation is an early event in PCa progression, as evident from a reduced level of SLC30A5 in stage II and grade group 1 samples only (Fig. 3c,d). This might be occurring transiently in PCa development, as there is no difference in stage III and in other higher-grade tumor samples. Similar to human PCa tissue data, SLC30A5 mRNA expression was downregulated in all tested PCa cell lines except MDA PCa 2b (Fig. 3e).

#### SLC30A6

SLC30A6, also known as ZnT6, is localized to the Golgi apparatus and *trans-*Golgi network^32^. Unlike other members of the Zn transporter family, SLC30A6 itself lacks the capability to transport Zn^40^, probably due to the absence of two of the four conserved hydrophilic residues in trans-membranes II and V, which are considered to be essential for both the formation of a Zn-binding site within the transmembrane domains and transportation of Zn across the cell membrane. However, SLC30A6 may function as a modulator of Zn transport activity through its participation in the SLC30A5/SLC30A6 heterodimer complex via the SLC30A6 Ser-rich loop. SLC30A6 localization is regulated by intracellular Zn levels and is redistributed from the perinuclear region to the cell periphery in response to high Zn levels^32^. SLC30A6 expression has been implicated in Alzheimer’s disease and is overexpressed in the hippocampus/parahippocampal gyrus and cerebellum of pre-clinical Alzheimer’s disease subjects^43^. However, SLC30A6 is downregulated in the spinal cords of patients with sporadic amyotrophic lateral sclerosis^44^. SLC30A6 is expressed uniformly across the prostate lobes during sexual maturation in mice^45^. SLC30A6 has not been studied in PCa.

**Figure 4:**
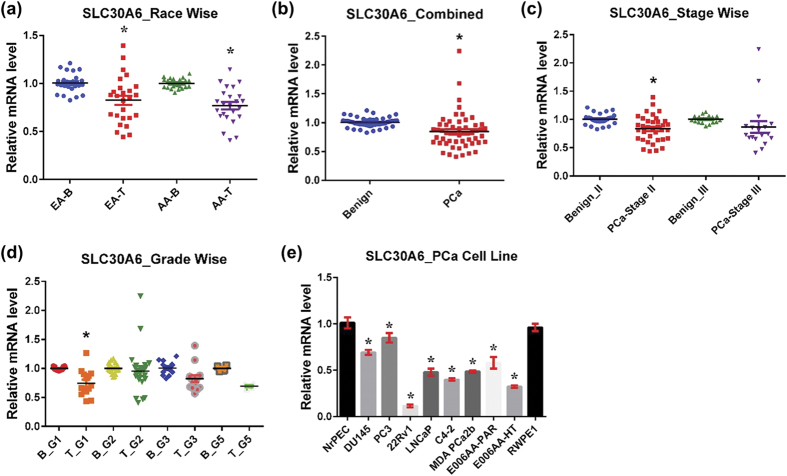
SLC30A6 in PCa. SLC30A6 mRNA levels were analyzed as detailed in Fig. 1. (**a**) Race-wise analysis of SLC30A6 in PCa. **(b)** SLC30A6 in combined PCa samples. **(c)** Tumor stage-wise modulation of SLC30A6 in PCa. **(d)** Tumor grade-wise modulation of SLC30A6 in PCa. **(e)** SLC30A6 in PCa cell lines.

We found an overall downregulation of SLC30A6 levels in PCa irrespective of EA and AA populations (Fig. 4a,b). Like SLC30A5, SLC30A6 downregulation seems to be an early event in PCa development as evident from a significant decrease in stage II and grade group 1 cancer samples (Fig. 4c,d). SLC30A6 expression was also downregulated in stage III and in other higher grade cancer samples (grade groups 3 and 5), but statistically not significantly. Congruently, SLC30A6 mRNA expression was downregulated in all human PCa cell lines (Fig. 4e).

#### SLC30A7

SLC30A7, also known as ZnT7, is localized to the early secretory pathway, primarily on the *cis*-face of the Golgi apparatus and cytoplasmic vesicles^46^,^47^. SLC30A7 is widely expressed, with abundant expression in the liver and small intestine and moderate expression in the brain, spleen, kidney, and lung^48^. SLC30A7 serves to transport Zn from the cytoplasm into the Golgi apparatus. Together with SLC30A5, SLC30A7 is responsible for loading Zn to alkaline phosphatases^48^. Thus, SLC30A7 and SLC30A5 are required for the activation of tissue non-specific alkaline phosphatase. SLC30A7 also plays a role in stimulating insulin synthesis and secretion through regulation of insulin gene transcription^49^.

SLC30A7 disruption in mice results in poor growth, reduced absorption of dietary Zn, and decreased body adiposity, indicating SLC30A7’s importance in dietary Zn absorption and body fat regulation^50^. Null-mutation in SLC30A7 is shown to accelerate prostate tumor formation in TRAMP mice, suggesting insufficient SLC30A7 activity may contribute to PCa progression^51^.

**Figure 5:**
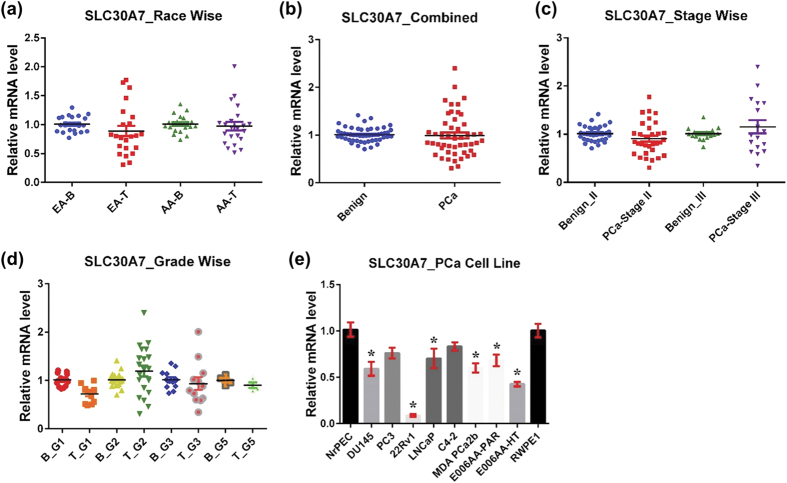
SLC30A7 in PCa. SLC30A7 mRNA levels were analyzed as detailed in Fig. 1. (**a**) Race-wise analysis of SLC30A7 in PCa. **(b)** SLC30A7 in combined PCa samples. **(c)** Tumor stage-wise modulation of SLC30A7 in PCa. **(d)** Tumor grade-wise modulation of SLC30A7 in PCa. **(e)** SLC30A7 in PCa cell lines.

In our study, we did not notice any difference in SLC30A7 mRNA expression between cancer and benign tissue in AA population and in the combined analysis (Fig. 5a,b). In addition, we observed a decreasing trend of SLC30A7 in PCa of EA samples; however, the data were not statistically significant. Interestingly, SLC30A7 was markedly downregulated in stage II and grade group 1 samples, upregulated in stage III and grade group 2 samples, and almost no change in group 3 and 5 samples (Fig. 5c,d). We further noticed a decline in SLC30A7 expression in all tested human PCa cell lines, though not statistically significant in PC3 and C4-2 cells (Fig. 5e).

#### SLC30A8

SLC30A8, also known as ZnT8, is primarily expressed in pancreatic α- and β-cells, but also found in other secretory cell types such as the epithelium of thyroid follicles and the adrenal cortex^52^,^53^. SLC30A8 supplies Zn to insulin secretory granules in β-cells to form insulin crystals, thereby contributing to the packaging efficiency and storage of insulin^54^. Pancreatic β-cells overexpressing SLC30A8 display enhanced glucose-stimulated insulin secretion in high glucose challenges, and deletion of SLC30A8 results in impaired insulin secretion^55^,^56^. Transcription of SLC30A8 is regulated by the pancreatic islet β-cell-enriched transcription factor Pdx-1 via an intronic enhancer^57^. Nonsynonymous single nucleotide polymorphism of SLC30A8 [SNP; rs13266634 (R325W)] confers type 2 diabetes risk^58^. This variant decreases Zn transport activity of SLC30A8^59^. SLC30A8 is also a major autoantigen in human type 1 diabetes (T1D), and is targeted by autoantibodies in 60–80% of new-onset T1D^60^. Overall, SLC30A8 seems to be a pancreas-specific Zn transporter, solely expressed in the secretory vesicles of β-cells and thus implicated in the final stages of insulin biosynthesis, which involve co-crystallization with Zn.

In our study, we found SLC30A8 mRNA expression too low to be detected in most of the samples. Specifically, we were able to detect SLC30A8 mRNA in few EA samples, and only in stage II and grade group 2 cancer (Supplemental Fig. 3a–d). In all these cases SLC30A8 expression was downregulated compared to their respective controls. A similar trend was noticed in PCa cell lines, where SLC30A8 was completely depleted, and therefore not detected in any of the PCa cells except negligible expression in DU145 (Supplemental Fig. 8e).

#### SLC30A9

SLC30A9, also known as ZnT9, is widely expressed in human tissue. It is localized in the cytoplasm and does not localize to the cell membrane^61^. SLC30A9 is believed to be localized in the nucleus as well. Interestingly, SLC30A9 is thought to lack Zn transport function due to the absence of an essential histidine involved in the intramembranous Zn-binding site. Furthermore, SLC30A9 does not interact with other SLC30A transporters, unlike SLC30A6. Originally, SLC30A9 was classified to the SLC30A transporter family based on its sequence similarity to the cation efflux domains of SLC30A members. Instead, SLC30A9 more accurately functions as a nuclear receptor coactivator and has taken the name GAC63 (GRIP1-associated coactivator)^62^. SLC30A9 plays a role in the p160 coactivator signaling pathway and mediates transcriptional activation via nuclear receptors. Additionally, SLC30A9 interacts with β-catenin and enhances its transcriptional activity in the Wnt signaling pathway^63^, whose activation is well known to affects PCa development and progression^64^.

**Figure 6:**
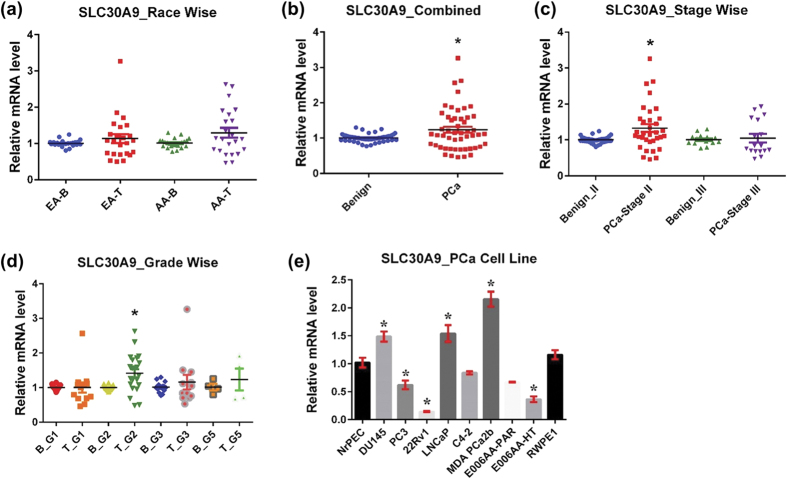
SLC30A9 in PCa. SLC30A9 mRNA levels were analyzed as detailed in Fig. 1. (**a**) Race-wise analysis of SLC30A9 in PCa. **(b)** SLC30A9 in combined PCa samples. **(c)** Tumor stage-wise modulation of SLC30A9 in PCa. **(d)** Tumor grade-wise modulation of SLC30A9 in PCa. **(e)** SLC30A9 in PCa cell lines.

We found markedly higher mRNA level of SLC30A9 in PCa tissue compared to adjacent normal tissue in both AA and EA samples. Interestingly, the increase was much more pronounced in AA specimens (Fig. 6a). Moreover, in the combined analysis, SLC30A9 was significantly overexpressed in PCa (Fig. 6b). Further analysis shows that SLC30A9 overexpression was limited to stage II tumors, as there was no change with stage III tumor samples (Fig. 6c). Also, there was no change in SLC30A9 expression in cancer grade group 1, but was significantly high in grade group 2 and markedly high in grade groups 3 and 5 (Fig. 6d). In PCa cell lines, SLC30A9 was significantly upregulated in DU145, LNCaP, and MDA PCa 2b, and downregulated in PC3, 22Rν1, and E006AA-HT. There was a decreasing trend of SLC30A9 in C4-2 and E006AA-Par cells, though statistically not significant (Fig. 6e).

#### SLC30A10

SLC30A10, also known as ZnT10, localizes in recycling endosomes and the Golgi apparatus^65^,^66^. In high extracellular Zn conditions, SLC30A10 is re-localized to the plasma membrane^65^. SLC30A10 is widely expressed, with the highest levels detected in the liver, brain, and small intestine tissues. The transportation activity of SLC30A10 is shown to be in the efflux direction, and functions to transport Zn into vesicles to prevent Zn toxicity^65^,^66^. SLC30A10 also exhibits important manganese (Mn) transporter function and serves to protect against intracellular Mn accumulation^67^,^68^,^69^. Homozygous mutation of SLC30A10 is known to cause Parkinsonism and dystonia with hypermanganesemia, polycythemia, and chronic liver disease and cirrhosis^67^,^68^. Interestingly, Mn and not Zn homeostasis was disturbed in these cases, suggesting SLC30A10’s primary function involves Mn transportation. Expression of SLC30A10 is found to be downregulated by IL-6 and angiotensin II, and downregulation of SLC30A10 by angiotensin II may be linked with cellular senescence in vascular smooth muscle cells^66^,^70^. SLC30A10 is known to be downregulated in the frontal cortex in Alzheimer’s disease, suggesting that dysregulation in SLC30A10 could further contribute to disease progression^71^. Additionally, SLC30A10 mRNA expression has been shown to be downregulated by Zn with reduced transcription from the SLC30A10 promoter at elevated extracellular Zn concentrations^65^.

**Figure 7:**
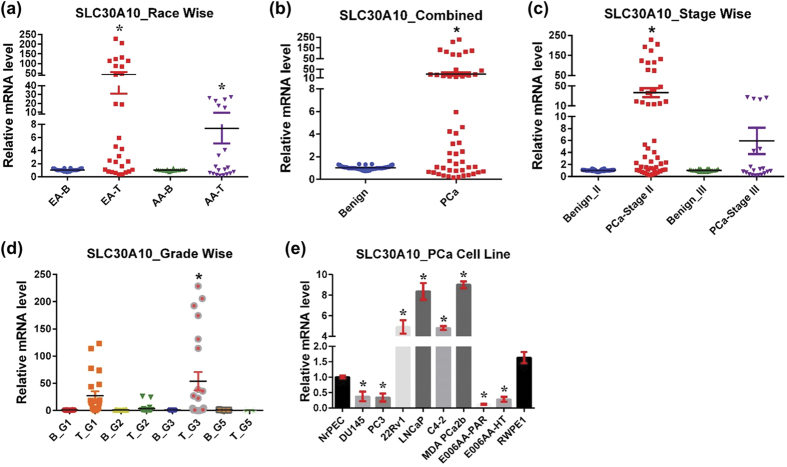
SLC30A10 in PCa. SLC30A10 mRNA levels were analyzed as detailed in Fig. 1. (**a**) Race-wise analysis of SLC30A10 in PCa. **(b)** SLC30A10 in combined PCa samples. **(c)** Tumor stage-wise modulation of SLC30A10 in PCa. **(d)** Tumor grade-wise modulation of SLC30A10 in PCa. **(e)** SLC30A10 in PCa cell lines.

In this study, we found that SLC30A10 mRNA was massively overexpressed in human PCa tissue, and too high in EA compare to AA samples (Fig. 7a,b). Though SLC30A10 is known to have functional activity in Zn efflux, how SLC30A10 overexpression relates to racial disparity remains unknown. High SLC30A10 expression was also noticed in stage II and III, and in grade groups 1 and 3 cancer (Fig. 7c,d). Moreover, SLC30A10 overexpression was noticed in PCa cell lines 22Rν1, LNCaP, C4-2, and MDA PCa 2b (Fig. 7e). Surprisingly, in the other four PCa cell lines (DU145, PC3, E006AA-Par, and E006AA-HT), SLC30A10 was downregulated.

### Oncomine mRNA data analysis of Zn-exporters

**Figure 8:**
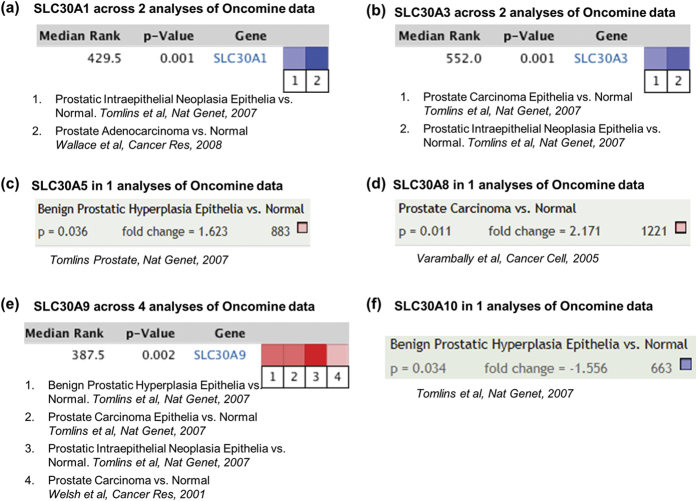
Oncomine Analysis of Zn-exporters in PCa. The oncomine datasets for respective Zn-exporters in PCa were retried at parameters p-value threshold of 0.05 with minimum 1.5-fold change. The data are shown in graphic that compares the number of datasets that had significant mRNA upregulation (red column) or downregulation (blue column) of the specified SLC30As in PCa versus normal tissue. The intensity of color shows the respective levels of SLC30As. **(a)** SLC30A1 across 2 analyses of Oncomine data set in PCa. **(b)** SLC30A3 in 1 analyses of Oncomine data set in PCa. **(c)** SLC30A5 in 1 analyses of Oncomine data set in PCa. **(d)** SLC30A8 in 1 analyses of Oncomine data set in PCa. **(e)** SLC30A9 in 4 analyses of Oncomine data set in PCa. **(f)** SLC30A10 in 1 analyses of Oncomine data set in PCa. No data sets were found for SLC30A2, SLC30A4, SLC30A6, and SLC30A7.

We further explored the expression of Zn-exporters transcripts in human tumor tissues using Oncomine, a cancer microarray database, which provides access to a large collection of cancer profiling datasets and analysis tools. We compared the data of clinical specimens of PCa versus normal and selected only those data showing ≥1.5-fold change with statistical significance (p = 0.05). We performed a comparative combined analysis where ever we found more than one datasets and presented average p-value. The Oncomine data for SLC30A2, SLC30A4, SLC30A6, and SLC30A7 in PCa were not found. Oncomine analysis for SLC30A1 showed significant levels of downregulation across two data sets, one comparing prostatic intraepithelial neoplasia epithelia versus normal, and another between prostate adenocarcinoma versus normal data (Fig. 8a). This is similar to our *in vitro* findings but contrary to human PCa tissue data. Further, an analysis of SLC30A3 across two Oncomine data sets revealed its downregulation (Fig. 8b), which is quite similar to the trend we observed in our limited data. For SLC30A5, the only one instance comparing benign prostatic hyperplasia with normal tissue was found, which revealed that SLC30A5 was upregulated (Fig. 8c). This data does not compare with PCa samples, thus not comparable with our data showing downregulation of SLC30A5 in PCa. Oncomine analysis showed SLC30A8 to be upregulated in PCa (Fig. 8d), which is in contrast to what we observed in our study. For SLC30A9, we found 4 data sets (including one comparing with prostatic hyperplasia), showing its upregulation in all the datasets, which is exactly similar to our findings (Fig. 8e). In Oncomine analysis for SLC30A10, no data was found comparing with PCa, however, we found a data set showing downregulation in benign prostatic hyperplasia compared to normal tissue (Fig. 8f).

### Ingenuity Pathway Analysis (IPA)

**Figure 9:**
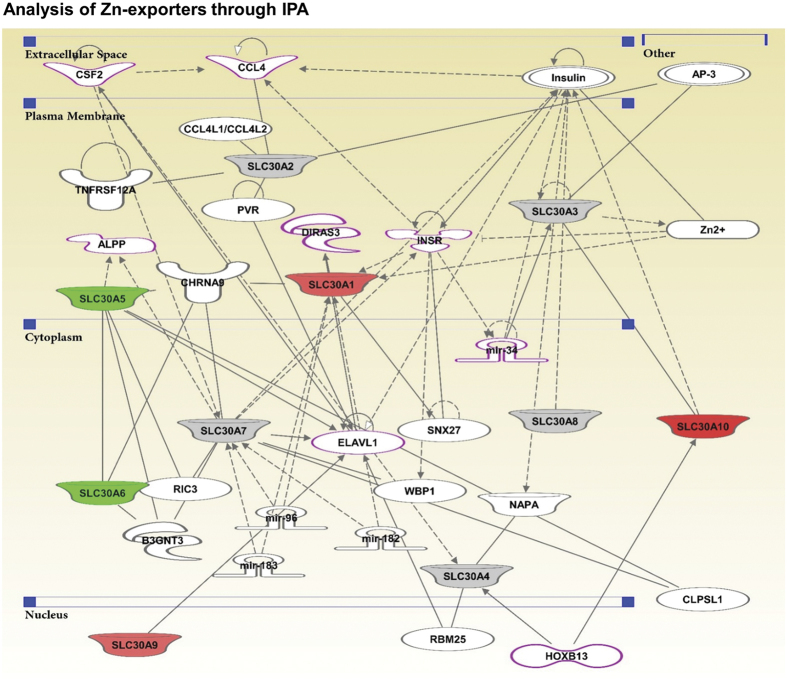
Ingenuity Pathway Analysis of differentially modulated SLC30As. Zn-exporters were subjected to IPA to generate gene-gene interaction networks. The highlighted molecules with pink boundary are those involved in PCa. The gene-gene interactions are indicated by arrows. The solid lines denote a robust correlation with partner genes, and dashed lines indicate statistically significant but less frequent correlations. The upregulated Zn-exporters are represented in red color, downregulated ones in green, and unchanged ones in gray color. The uncolored nodes indicate additional genes of this network that appeared to complete Zn-exporter pathways.

The differentially modulated Zn-exporters (SLC30As) were added to IPA to generate network pathways (Fig. 9). IPA, based on structured content from Ingenuity’s Pathways Knowledge Base (IPKB) transforms genes of interest into a set of relevant networks. Through IPA, we identified regulatory relationships between Zn-exporters and key tumor promoter/suppressor genes known to be involved in PCa development and progression. IPA revealed a direct interaction of HOXB13, a specific marker for benign and neoplastic prostate tissue, with SLC30A10, which we found significantly upregulated in PCa (Fig. 9). HOXB13, a homeobox protein and a key regulator of the epithelial differentiation in the prostate gland, is known to be overexpressed during malignant progression and thought to contribute to prostate carcinogenesis^72^. Interestingly, HOXB13 also shows a robust correlation with SLC30A4, which appears unchanged in PCa. SLC30A9, which we also found upregulated in this study, appears to regulate ELAVL1, an RNA-binding protein known to be involved in the regulatory process of PCa progression. Recently, Melling and colleagues have identified cytoplasmic accumulation of ELAVL1 as a predictor of adverse clinical behavior of PCa^73^. Additionally, SLC30A1, which we found overexpressed in PCa, shows direct interaction with DIRAS3, also known as Guanosine-5′-triphosphate (GTP)-binding RAS-like 3 (ARHI), which is a tumor suppressor and known to be downregulated in PCa compared with adjacent normal tissues^74^. In addition, IPA analysis explored several important interactions with other Zn-exporters, such as a robust interaction of SLC30A6 (which is downregulated) with CHRNA9, B3GNT3, as well as with SLC30A5, which itself is downregulated in PCa.

## Conclusions

We have provided evidence that Zn-exporter proteins may be involved in the racial disparities in PCa in AA versus EA. The plausible explanations for this phenomenon is the high Zn concentrations in the soil and water in mineral-rich African continent^75^, and/or differential natural selection of Zn transporter genes in African population^76^. Therefore, individuals with African ancestry may have a natural adaptation for low Zn accumulation to avoid Zn toxicity by adjusting the levels of Zn-importer (SLC39As) or Zn-exporter (SLC30As) or both. We have found that mRNA levels of SLC30A1, SLC30A9 and SLC30A10 were significantly high in PCa tissue compared to adjacent normal tissue. However, SLC30A5 and SLC30A6 were significantly downregulated (Fig. 9). Interestingly, significant upregulation of mRNA levels of SLC30A1 and SLC30A10, and marked downregulation of SLC30A7 mRNA levels were more pronounced in EA PCa specimens than AA. On the other hand, while there was no change in the levels of SLC30A1 and SLC30A7 between normal and PCa in AA patients, a markedly pronounced upregulation was observed for SLC30A9 compared to the increase observed in EA patients. These observations are important in further delineating the mechanism of the observed PCa racial disparity in AA vs EA population.

Our study also showed that Zn transporters SLC30A2, SLC30A3 and SLC30A8 were not detectable in several human PCa tissues samples. Thus, their role remains inconclusive, based on our limited investigation. At the mRNA levels SLC30A4 and SLC30A7 did not show any change between benign and tumor samples. The differential expression of SLC30A1, SLC30A5, SLC30A6, SLC30A9 and SLC30A10 was found to vary with tumor stage and grade and appears to be related mostly to early events in PCa development and progression. Interestingly, high variability for Zn-exporters expression was noticed among the PCa cell lines with varying degrees of genetic complexity, and in some cases, the cell line results did not match the human tissue PCa data. However, the variability among Zn-exporters does not correlate with the characteristics of the cell lines as detailed in Supplementary Table 1. These apparent inconsistencies between the data sets of human PCa tissue and *in vitro* cell line data demand further investigation. Additionally, Oncomine analysis of existing data showed a contrasting result for SLC30A1 but was strongly supportive for SLC30A9. Oncomine data sets were not available for SLC30A2, SLC30A4, SLC30A6, and SLC30A7, and not comparable for SLC30A3, SLC30A5, SLC30A8 and SLC30A10. Furthermore, our study identified the potential interaction of several tumor suppressors and promoters (HOXB13, ELAVL1, DIRAS3, CHRNA9, B3GNT3, RIC3, ALPP, CSF2, CCL4, and INSR) with the significantly modulated Zn-exporters (SLC30A1, SLC30A5, SLC30A6, SLC30A9, and SLC30A10). Thus, our study presents interesting data regarding the expression profiles of Zn-exporters and their interaction with other important genes in PCa.

## Methods

### Cell culture

Human PCa cell lines (DU145, 22Rν1, PC3, LNCaP, C4-2 and MDA PCa 2b) were obtained from American Type Culture Collection (ATCC) and maintained in RPMI-1640 supplemented with 10% FBS at standard cell culture conditions. PCa cells E006AA-Par and E006AA-hT were received as a kind gift from Dr. Shahriar Koochekpour (Roswell Park Cancer Institute) and maintained in DMEM plus 10% FBS. Normal prostate epithelial cells (NrPEC) were obtained from ATCC and maintained in prostate epithelial cell basal medium supplemented with L-Glutamine, Extract P, Epinephrine, rh TGF-α, Hydrocortisone hemisuccinate, rh Insulin and Apo-transferrin as per ATCC instructions. Similarly, RWPE-1, a virally immortalized epithelial cell line, was also obtained from ATCC and maintained per ATCC instructions in Keratinocyte Serum Free Medium (K-SFM), (Invitrogen (GIBCO)) supplemented with bovine pituitary extract and human recombinant epidermal growth factor. Characteristics of all these cell lines are detailed in Supplementary Table 1. Before experimentation, these cells were regularly tested for mycoplasma using the Mycoplasma Detection Kit (Lonza) according to manufacturer’s protocol, with fluorescence readings done on a Synergy H1 multimode microplate reader (BioTek). These cells were grown under standard cell culture conditions, and cells were collected at ~80% confluency to be used for RNA isolation.

### qRT-PCR analysis with PCa cell line samples

RNA was extracted from the cells using QIAshreder and RNeasy Plus Mini Kit (Qiagen), quantified using Synergy H1 BioTek microplate reader, and first strand cDNA was transcribed with oligo(dT), dNTPs and M-MLV reverse transcriptase (Promega). qRT-PCR was performed using StepOnePlus Real-Time PCR system (Life Technologies) and Platinum SYBR Green qPCR SuperMix-UDG (Takara). Primers for Zn-exporters and GAPDH were selected from PrimerBank database^77^, as detailed in Supplementary Table 2. Relative quantification for the gene of interest was analyzed using GAPDH as endogenous control and ΔΔC_T_ algorithm.

### qRT-PCR analysis with human PCa tissue samples

Prostatectomy samples from AA and EA men, with known cancer grades and stages, were obtained from Dr. Kenneth lczkowski, a urologic/GU pathologist at the Medical College of Wisconsin. Tissue blocks were procured under the supervision of Dr. lczkowski with institutional review board (IRB) approval (PRO00023514). Hematoxylin-eosin stained slides were marked according to non-hyperplastic benign areas and tumor areas. The corresponding benign and tumor areas on unstained slides were scratched out with sterile surgical blades and processed for RNA isolation using RNeasy formalin fixed paraffin-embedded (FFPE) kit (Qiagen) per manufacturer’s protocol. This kit is designed for efficiently extracting RNA from tissue sections by reversing formaldehyde modification of RNA. The quality and quantity of isolated RNA were assessed using Synergy H1 hybrid multi-mode microplate reader (BioTek). First strand cDNA was transcribed with random primers, dNTPs and M-MLV reverse transcriptase (Promega). The qRT-PCR analysis of Zn-exporters was performed as described above.

### Oncomine data analysis

The expression level of Zn-exporters in PCa was also analyzed using Oncomine (https://www.oncomine.org/resource/login.html), a web-based data-mining platform aimed at facilitating discovery from genome-wide expression analyses^78^. The Oncomine datasets were obtained at the parameters: threshold p-value of 0.05 with minimum 1.5-fold change. Datasets that had significant mRNA overexpression or underexpression of the specified Zn-exporters in PCa *versus* benign tissue were selected for the analysis.

### Zn-exporters gene network analysis

To understand pathways controlled by Zn-exporters, an Ingenuity Pathway Analysis (IPA, Qiagen) was performed with differentially modulated SLC30As. The predicted gene-gene interaction network was generated with highlights of genes known to be modulated in PCa.

### Statistical analysis

For statistical purposes, cancer Gleason scores were compressed into the 5-tier prognostic grade grouping system endorsed by the International Society for Urologic Pathology and World Health Organizatio^12^. Gleason 3 + 3 is taken as grade group 1, 3 + 4 is group 2, 4 + 3 is group 3, Gleason score 8 is group 4, and Gleason scores 9–10 are group 5. qRT-PCR data were analyzed using StepOne Software v2.2 RQ Study (Life Technologies Corp.) and exported as RQmax and RQmin (2^−ΔΔCt +/− ΔΔCt SD^) which represent relative quantities for the gene of interest. RQmax and RQmin are the results of incorporating standard deviation of the ΔΔC_T_ into the fold-change calculations. Further, statistical analyses on biological replicates of qRT-PCR data were performed with GraphPad Prism 5 software (GraphPad Software Inc.) using one-way analysis of variance (ANOVA) followed by Sidak/Dunnett’s multiple comparison tests. Two-tailed unpaired t-test was done for combined benign and tumor samples.

## Additional Information

**How to cite this article**: Singh, C. K. *et al*. Analysis of Zinc-Exporters Expression in Prostate Cancer. *Sci. Rep.* **6**, 36772; doi: 10.1038/srep36772 (2016).

**Publisher’s note:** Springer Nature remains neutral with regard to jurisdictional claims in published maps and institutional affiliations.

## Supplementary Material

Supplementary Information
