# Supplementary material for: Analysis of Zinc-Exporters Expression in Prostate Cancer
Source: Sci Rep. 2016 Nov 11;6:36772. doi: 10.1038/srep36772 (PMC5105060; doi:10.1038/srep36772)
Supplement: Supplementary Information [file srep36772-s1.doc]

**Analysis of Zinc-Exporters Expression in Prostate Cancer**

Chandra K. Singh^1*^, Kareem M. Malas^1^, Caitlin Tydrick^1^, Imtiaz A. Siddiqui^1^, Kenneth A. Iczkowski^2^, Nihal Ahmad^1^

***Supplemental Figures***

**Supplemental Figure 1: SLC30A2 in PCa.** SLC30A2 mRNA levels were analyzed as detailed in figure 1. **(A)** Race-wise analysis of SLC30A2 in PCa. **(B)** SLC30A2 in combined PCa samples. **(C)** Tumor stage-wise modulation of SLC30A2 in PCa. **(D)** Tumor grade-wise modulation of SLC30A2 in PCa. **(E)** SLC30A2 in PCa cell lines.

**Supplemental Figure 2: SLC30A3 in PCa.** SLC30A3 mRNA levels were analyzed as detailed in figure 1. **(A)** Race-wise analysis of SLC30A3 in PCa. **(B)** SLC30A3 in combined PCa samples. **(C)** Tumor stage-wise modulation of SLC30A3 in PCa. **(D)** Tumor grade-wise modulation of SLC30A3 in PCa. **(E)** SLC30A3 in PCa cell lines.

**Supplemental Figure 3: SLC30A8 in PCa.** SLC30A8 mRNA levels were analyzed as detailed in figure 1. **(A)** Race-wise analysis of SLC30A8 in PCa. **(B)** SLC30A8 in combined PCa samples. **(C)** Tumor stage-wise modulation of SLC30A8 in PCa. **(D)** Tumor grade-wise modulation of SLC30A8 in PCa. **(E)** SLC30A8 in PCa cell lines.

***Supplemental Tables***

**Supplemental Table 1**: Characteristics of prostate cancer cell lines and status of zinc exporters analyzed therein.

| Cell line | Ethnic background | Origin | Tumorigenic potential | P53 status | PSA status | Androgen sensitivity | Androgen receptor (AR) | Zinc exporters (SLC30A) |
| --- | --- | --- | --- | --- | --- | --- | --- | --- |
| DU145 | Caucasian male, 69- year-old | Derived from metastatic site: brain | Moderate | P53 mutated | No | Insensitive | AR negative | **1, 2, 3, 4, 5, 6, 7, 8, 9, 10** |
| 22Rν1 | Caucasian | Derived from a human prostate tumor xenografts CWR22R | Moderate | P53+ | Yes | Sensitive | AR positive | **1, 2, 3, 4, 5, 6, 7, 8, 9, 10** |
| PC3 | Caucasian, male, 62- year-old | Bone metastasis grade IV of prostate cancer | High | P53 null/- | No | Insensitive | AR negative | **1, 2, 3, 4, 5, 6, 7, 8, 9, 10** |
| LNCaP | Caucasian  male, 50- year-old | Derived from metastatic supra-clavicular lymph node | Very low | P53+ | Yes | Sensitive | AR mutated | **1, 2, 3, 4, 5, 6, 7, 8, 9, 10** |
| C4-2 | Caucasian | Derived from LNCaP cells | Moderate | P53 low level | Yes | Insensitive | AR positive | **1, 2, 3, 4, 5, 6, 7, 8, 9, 10** |
| MDA PCa 2b | African American male, 63-year-old | Derived from metastatic site: bone | High | P53+ | Yes | Sensitive | AR positive | **1, 2, 3, 4, 5, 6, 7, 8, 9, 10** |
| E006AA-Par | African American, male, 50-year-old | Derived from pathologically  confined primary prostatic tumors | Limited tumorigenicity | P53+ | Low | Sensitive | AR positive | **1, 2, 3, 4, 5, 6, 7, 8, 9, 10** |
| E006AA-hT | African American | Derived from E006AA cells | High | P53+ | Low | Sensitive | AR positive | **1, 2, 3, 4, 5, 6, 7, 8, 9, 10** |
| NrPEC | Caucasian male, 22- year-old | Normal prostate epithelial cells | Non- tumorigenic | P53+ | Low | AR expression in NrPEC is associated with differentiation | | **1, 2, 3, 4, 5, 6, 7, 8, 9, 10** |
| RWPE1 | Caucasian male, 54- year-old | Immortalized normal prostate epithelial cells | Non- tumorigenic | P53+ | Secrete PSA when exposed to androgen | | AR positive | **1, 2, 3, 4, 5, 6, 7, 8, 9, 10** |

**Note:** Red marked indicate downregulation, blue indicate upregulation and black as no change compared to NrPEC.

**Supplemental Table 2**: Primer sequences used in this study for Zn exporters analysis.

| Gene | Amplicon size (bp) | Primer orientation | Primer Sequence (5' - > 3') | Length | Tm | Primer Bank ID |
| --- | --- | --- | --- | --- | --- | --- |
| SLC30A1 | 128 | F | GGACAACTTAACATGCGTGGA | 21 | 60.6 | 52352802c3 |
|  |  | R | ACACAAAAATCCCCTTCAGAACA | 23 | 60.3 |  |
| SLC30A2 | 122 | F | TGTCCTAGTGGCAGCCTATATT | 20 | 60.1 | 52352806c3 |
|  |  | R | TCACATCTCTCAGGATGGTCAA | 20 | 60.2 |  |
| SLC30A3 | 198 | F | CACCCGCACCATGACCTTT | 19 | 62.6 | 52630414c2 |
|  |  | R | AAGGCCATTAACAGGTTGGCA | 21 | 62.3 |  |
| SLC30A4 | 106 | F | TGACCTAAGCGCCATCATACT | 21 | 60.7 | 52630415c3 |
|  |  | R | AGCTGACAAAACCTCTAAGCG | 21 | 60.3 |  |
| SLC30A5 | 108 | F | ACCAAACACCAGTGGATCAAAA | 22 | 60.0 | 20070323a1 |
|  |  | R | CAGCAAAGTCCTTAGTGGTCC | 21 | 60.3 |  |
| SLC30A6 | 126 | F | AGGAAACCTAGCCCTGTCTATT | 22 | 60.3 | 301898445c1 |
|  |  | R | CAAAAAGCGTTCTGCACTTTCT | 22 | 60.2 |  |
| SLC30A7 | 183 | F | TCTCTTTCGCTTTTGTGGAACT | 22 | 60.1 | 222080084c2 |
|  |  | R | CCAGAACTTCCGCTCTAACATAC | 23 | 60.4 |  |
| SLC30A8 | 116 | F | GATCCAGGCGACTGTGATGAT | 21 | 61.7 | 289803012c3 |
|  |  | R | TGGCTTGTACTTCCTTGTGATTG | 23 | 60.7 |  |
| SLC30A9 | 172 | F | TCAGGGTTTACTAGCATTGGGC | 22 | 62.0 | 57164947c3 |
|  |  | R | AGGATGAAGCAATCCCATGACT | 22 | 61.2 |  |
| SLC30A10 | 80 | F | GCTGTGCCTGGAATTAGCAGT | 21 | 62.7 | 52351207c3 |
|  |  | R | ATGTGCAGGGTGGCAATAATC | 21 | 60.7 |  |
| GAPDH | 101 | F | CTGGGCTACACTGAGCACC | 19 | 62.0 | 378404907c3 |
|  |  | R | AAGTGGTCGTTGAGGGCAATG | 21 | 62.9 |  |
